# Extensive lipid lowering, thickness of the fibrous caps, and plaque stability

**DOI:** 10.1093/ehjopen/oeae056

**Published:** 2024-07-03

**Authors:** Shinya Goto, Shinichi Goto

**Affiliations:** Department of Medicine, Tokai University School of Medicine, 143 Shimokasuya, Isehara, Kanagawa 259-1193, Japan; Department of Medicine, Tokai University School of Medicine, 143 Shimokasuya, Isehara, Kanagawa 259-1193, Japan

**This editorial refers to ‘Early and short-term use of proprotein convertase anti-subtilisin–kexin type 9 inhibitors on coronary plaque stability in acute coronary syndrome’, by H. Uehara *et al*., https://doi.org/10.1093/ehjopen/oeae055.**

There is extensive clinical evidence demonstrating the prevention of acute coronary events, including myocardial infarction, by lowering the LDL-cholesterol using potent statins.^1^ Recently developed proprotein convertase subtilisin/kexin 9 (PCSK9) inhibitor further reduces LDL cholesterol and the risk of future cardiovascular (CV) events.^2^ The precise mechanism of the action of lipid-lowering drugs to reduce the risk of future CV events is still to be elucidated. Stabilization of lipid plaques in coronary arteries might be one of the contributing factors.^3^ In this issue of European Heart Journal Open, Uehara *et al*. compared the increase in minimum fibrous cap thickness (MFCT) detected by optical coherent tomography (OCT) in the coronary arteries from the baseline between those treated by statin alone and those with statin + PCSK9 inhibitor.^4^ They showed that the magnitude of increase was larger in patients on statin alone compared with those treated by statin + PCSK9 inhibitor at 9-month period. The authors concluded that ‘Combination treatment with PCSK9Is and statins resulted in more marked plaque stabilization after ACS than SoC alone’. However, it should be noted that the authors only showed the thickening of the so-called fibrous cap detected by OCT. The authors did not show any evidence that the plaques in patients treated by the combination of PCSK9 inhibitor and statin stabilized.

The authors attempted to quantify the MFCT from the image obtained by the OCT. The OCT images are believed to be more clear as compared with the previously established ultrasonography. However, the image resolution quality of OCT still is not high enough to detect the shape of endothelial cells^5^ (*Figure 1*). The OCT images obtained in this study may not reflect histologically defined components of vessel wall such as intima, media, and adventitia. Despite the limitation, the authors’ efforts to obtain the OCT image serially at baseline, 3 months, and 9 months should still be commended. Since the standardization of the quantification of OCT images is not easy, these serially recorded images should be better standardized than those obtained in multicentre studies. While the results presented by the authors might not completely reflect the histology, it might still show some signals related to the use of potent lipid-lowering therapy.

**Figure 1:**
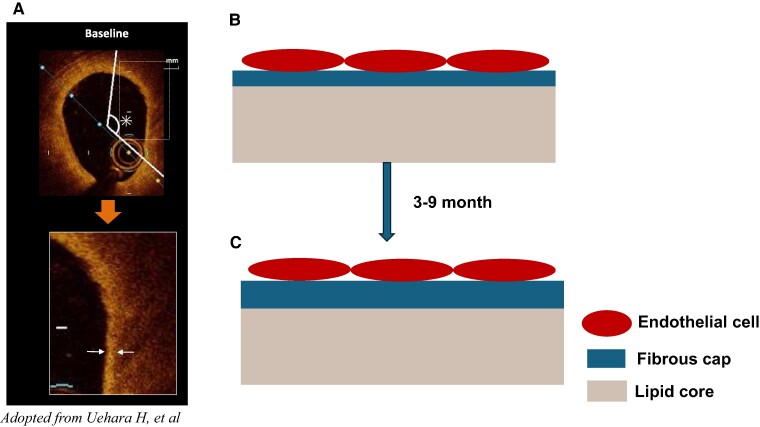
Application of optical coherence tomography for detecting fibrous cap in coronary arterial plaque. In this paper, the authors Uehara H et al. attempted to detect the thickness of the fibrous cap covering the lipid core. (*A*) Adopted from the original article’s left panel of *Figure 4*. The authors aimed to support their hypothesis that the fibrous cap becomes thicker with strong lipid-lowering therapy as shown in (*B*) and (*C*). However, image resolution quality of OCT is not high enough to separate endothelial cells. Thickening of fibrous cap detected by OCT images might not reflect the stability of the plaque. Adopted from Uehara H, *et al.*^4^

In addition to serially recorded OCT images, this paper provides some potentially important information. The doses of statin used in the East Asian countries are lower compared with the other regions of the world.^6^ All participants in this study received 20 mg of atorvastatin, which is the maximum dose approved locally. Interestingly, the author-defined MFCT increased during the observation period, even in patients not receiving PCSK9 inhibitor. This result may suggest that higher doses of statin should be used even in East Asian countries to avoid CV events. So far, no clear evidence shows the relationship between MFCT and future risk of CV events. If the authors could show the relationship between MFCT and the future risk of major cardiovascular events with a more extensive sample-sized study in the future, MFCT might be a good surrogate marker for screening future lipid-modifying agents such as Lipoprotein(a) inhibitors.

Currently, the MFCT could be measured only by interventional cardiologists using OCT. If the clinical impacts of measuring MFCT were established in the future, various novel technologies could be developed to detect MFCT from the outside of the body. The current study by Uehara *et al*. has a lot of limitations, but future studies to clarify the clinical impacts of MFCT are awaited.

## Data Availability

Our opinions are based upon the data provided as https://doi.org/10.1093/ehjopen/oeae055. We do not have any original data to add.

## References

[1] Cannon CP, Steinberg BA, Murphy SA, Mega JL, Braunwald E. Meta-analysis of cardiovascular outcomes trials comparing intensive versus moderate statin therapy. J Am Coll Cardiol 2006;48:438–445. 16875966 10.1016/j.jacc.2006.04.070

[2] Sabatine MS, Giugliano RP, Keech AC, Honarpour N, Wiviott SD, Murphy SA, Kuder JF, Wang H, Liu T, Wasserman SM, Sever PS, Pedersen TR; FOURIER Steering Committee and Investigators. Evolocumab and clinical outcomes in patients with cardiovascular disease. N Engl J Med 2017;376:1713–1722. 28304224 10.1056/NEJMoa1615664

[3] Vogel B, Claessen BE, Arnold SV, Chan D, Cohen DJ, Giannitsis E, Gibson CM, Goto S, Katus HA, Kerneis M, Kimura T, Kunadian V, Pinto DS, Shiomi H, Spertus JA, Steg PG, Mehran R. ST-segment elevation myocardial infarction. Nat Rev Dis Primers 2019;5:39. 31171787 10.1038/s41572-019-0090-3

[4] Uehara H, Kajiya T, Abe M, Nakata M, Hosogi S, Ueda S. Early and short-term use of proprotein convertase anti-subtilisin–kexin type 9 inhibitors on coronary plaque stability in acute coronary syndrome. Eur Heart J Open 2024;4. doi:10.1093/ehjopen/oeae055. PMC11316204 39131906

[5] Huang D, Swanson EA, Lin CP, Schuman JS, Stinson WG, Chang W, Hee MR, Flotte T, Gregory K, Puliafito CA. Optical coherence tomography. Science 1991;254:1178–1181. 1957169 10.1126/science.1957169 PMC4638169

[6] Torii S, Chiang C-E, Hong SJ, Goto S, Huang W-C, Chan MY-Y, Kajiya T, Goto S. Asian perspective on the recently published practice guideline for acute coronary syndrome by ESC. Eur Heart J Acute Cardiovasc Care 2024;13:162–164. 37832510 10.1093/ehjacc/zuad126

